# Accuracy of the first interpretation of early brain CT images for predicting the prognosis of post-cardiac arrest syndrome patients at the emergency department

**DOI:** 10.1186/s40560-018-0296-6

**Published:** 2018-04-25

**Authors:** Mitsuaki Nishikimi, Takayuki Ogura, Kota Matsui, Kunihiko Takahashi, Kenji Fukaya, Keibun Liu, Hideo Morita, Mitsunobu Nakamura, Shigeyuki Matsui, Naoyuki Matsuda

**Affiliations:** 1 Department of Emergency and Critical Care, Nagoya University Graduate School of Medicine, Tsurumai-cho 64, Syowa-ku, Nagoya, Aichi 466-8560 Japan; 2 Advanced Medical Emergency Department and Critical Care Center, Japan Red Cross Maebashi Hospital, Maebashi, Japan; 3 Department of Biostatistics, Nagoya University Graduate School of Medicine, Nagoya, Japan; 4 Department of Diagnostic Radiology, Japan Red Cross Maebashi Hospital, Maebashi, Japan

**Keywords:** Cardiac arrest, Post-cardiac arrest syndrome, Neurological prognosis, Brain CT scan, Targeted temperature management

## Abstract

**Background:**

Early brain CT is one of the most useful tools for estimating the prognosis in patients with post-cardiac arrest syndrome (PCAS) at the emergency department (ED). The aim of this study was to evaluate the prognosis-prediction accuracy of the emergency physicians’ interpretation of the findings on early brain CT in PCAS patients treated by targeted temperature management (TTM).

**Methods:**

This was a double-center, retrospective, observational study. Eligible subjects were cardiac arrest patients admitted to the intensive care unit (ICU) for TTM between April 2011 and March 2017. We performed the McNemar test to compare the predictive accuracies of the interpretation by emergency physicians and radiologists and calculated the kappa statistic for determining the concordance rate between the interpretations by these two groups.

**Results:**

Of the 122 eligible patients, 106 met the inclusion criteria for this study. The predictive accuracies (sensitivity, specificity) of the interpretations by the emergency physicians and radiologists were (0.34, 1.00) and (0.41, 0.93), respectively, with no significant difference in either the sensitivity or specificity as assessed by the McNemar test. The kappa statistic calculated to determine the concordance between the two interpretations was 0.66 (0.48–0.83), which showed a good conformity.

**Conclusions:**

The emergency physicians’ interpretation of the early brain CT findings in PCAS patients treated by TTM was as reliable as that of radiologists, in terms of prediction of the prognosis.

## Background

One of the most important clinical considerations in patients with post-cardiac arrest syndrome (PCAS) is to estimate the neurological prognosis [1, 2]. A definitive estimation of the prognosis of PCAS patients undergoing targeted temperature management (TTM) should be performed 72 h after the return to normal body temperature according to the guideline [3]. But a few previous studies have reported the usefulness of early estimation of the prognosis in PCAS patients at the time of the arrival at the emergency department (ED) [4–6].

Early brain CT is one of the most useful tools for estimating the prognosis of PCAS patients at the ED [7]. Although a few small studies have shown that signs of loss of gray-white matter differentiation and brain swelling on brain CT are reliable signs of a poor prognosis, the interpreters in these studies were imaging specialists, or radiologists, and not emergency physicians [8–11]. Considering that it is impossible for radiologists to evaluate the CT scans in real time at the ED in many countries [12, 13], there is no doubt about the importance of accurate interpretation by emergency physicians. But no study has been conducted to determine the accuracy of interpretation of early brain CT images by emergency physicians for estimating the prognosis in PCAS patients. Thus, the aim of this study was to evaluate the accuracy of interpretation of early brain CT by emergency physicians in comparison with that by expert radiologists in PCAS patients treated by TTM.

## Methods

### Study design

A double-center, retrospective, observational study was performed. We retrospectively reviewed the clinical management charts of the patients with cardiac arrest admitted to the Nagoya University Hospital or Japan Red Cross Maebashi Hospital between April 2011 and March 2017. All eligible patients were more than 20 years old and had lived independently prior to the development of the cardiac arrest. Subjects were included in this study if they had undergone a brain CT at the ED after return of spontaneous circulation (ROSC) following cardiac resuscitation, and these CT images had been interpreted by both the emergency physicians at the ED and radiologists. Note that a brain CT examination is routinely performed for PCAS patients before the initiation of TTM in these two hospitals. After the admission, TTM was undertaken in all the patients at the intensive care unit (ICU) by cold infusion and a surface cooling device with computerized automatic temperature control.

### Dataset

Data were collected retrospectively by reviewing the electronic medical charts of the patients, including the clinical histories, cardiac rhythms, physical examination findings, blood examination results, brain CT image findings, and clinical courses after admission.

To compare the interpretations of the CT images by the emergency physicians and radiologists, we conducted a retrospective review of the records of the findings of the emergency physicians and reports of the radiologists. We only reviewed those findings that had been entered by the emergency physicians before the radiologists’ reports became available, so as to exclude the possibility of the latter influencing the interpretation by the emergency physicians. When phrases such as “signs of loss of gray-white matter differentiation or brain swelling was seen” were found in the records, we judged that the interpreter had recognized the signs of the hypoxic encephalopathy. At the two participant hospitals, emergency physicians at the ED must interpret the findings on early brain CT while having no access to the reports by radiologists, because the radiologists provide their reports only after (within 2 days) the emergency physicians’ interpretation. The emergency physicians in this study were defined as specialists and fellows working on a regular basis at our ED, and all of them had the experience of working at the ED as residents for at least 2 years.

### The calculation of the gray matter attenuation to white matter attenuation ratio

One blinded critical care fellow calculated the gray matter attenuation to white matter attenuation ratio (GWR) by retrospectively reviewing the CT images of all subjects. It was measured using the method described in Torbey et al.’s report [14]. The GWR was compared with the predictive accuracy of the interpretation by the emergency physicians in order to validate the latter with objective indices. The intensities of circular areas of interest (about 10 mm2) were measured for both the gray and white matter on three axial slices (5-mm slice thickness) at a basal ganglia level, a centrum semiovale level, and a high convexity level. Then, the GWR was calculated as shown below:

GWR basal ganglia = [PU + CN]/[CC + PIC]

GWR cortex = [MC1 + MC2]/[MWM1 + MWM2]

GWR = [GWR basal ganglia + GWR cortex]/2

where PU indicates putamen, CN caudal nucleus, CC corpus callosum, PIC posterior limb of internal capsule, MC1 medial cortex at centrum semiovale, MC2 medial cortex at high convexity level, MWM1 medial white matter at centrum semiovale, and MWM2 medial white matter at high convexity level. Each value was the average of the right and left hemisphere values.

### Protocol for targeted temperature management

TTM was undertaken in the eligible patients according to the protocol in place at each of the hospitals. TTM was considered as being indicated for cardiac arrest patients who were in a coma (GCS ≤ 8) after ROSC without remarkable hemodynamic instability or a “Do-Not-Attempt to Resuscitate” directive. The temperature was maintained at the target level of 34–36 °C by infusion of cold fluids in combination with surface cooling with an ice pack and/or a cold blanket or using a surface cooling device with computerized automatic temperature control (Arctic Sun 2000 TTM; Bard Medical Louisville, CO). After the targeted temperature had been maintained for 24 h, rewarming to 36 °C was performed at the rate of 0.2 °C/4 h at Nagoya University Hospital or 1.0 °C/24 h at Japan Red Cross Maebashi Hospital. Propofol, midazolam, dexmedetomidine, fentanyl, and rocuronium were used for sedation, analgesia, and muscle relaxation, according to individual clinician preferences.

### Neurological outcome

The Cerebral Performance Categories (CPC) at 30 days was used to estimate the neurological outcome as follows: CPC 1, full recovery; CPC 2, moderate disability; CPC 3, severe disability; CPC 4, coma or vegetative state; and CPC 5, death [15]. CPC 1 and CPC 2 were considered as representing a good outcome, and CPC 3, CPC 4, and CPC 5 were considered as representing a poor outcome.

### Statistical analysis

For outcome, we derived the sensitivity and specificity of the interpretations by the emergency physicians and radiologists. We performed McNemar’s test to compare the sensitivity and specificity of the two interpretations. Next, in order to investigate the concordance rate between the two interpretations, we calculated the kappa statistic and its 95% confidence interval. All the statistical analyses were conducted using R software version 3.3.1 [16].

## Results

**Fig. 1:**
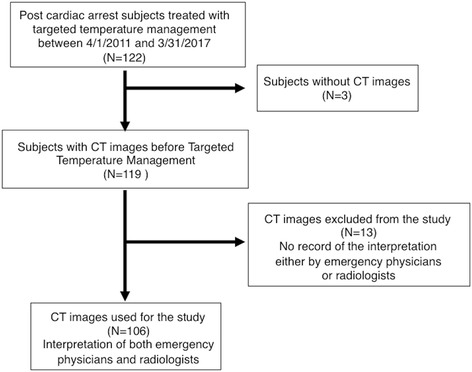
Subjects included in the study

During the study period, 122 PCAS patients were admitted to the ICU at either of the participant institutions for TTM, of whom 119 had undergone early brain CT prior to the initiation of the TTM. Of these 119 patients, 13 were excluded because of the lack of availability of the records of CT image interpretation by the emergency physicians and/or radiologists, and the remaining 106 were included in this study (Fig. 1).

The baseline characteristics of the subjects are summarized in Table 1. TTM at 34–36 °C was undertaken for all the patients at the ICU by infusion of cold fluids and use of a surface cooling device with computerized automatic temperature control. Most of the subjects were male (82.1%), with a median age of 64.0 (52.0–71.0) years and median hospital stay of 29.0 (19.0–54.0) days. Forty-five subjects (42.5%) showed good outcomes, while 61 subjects (57.5%) showed poor outcomes.

**Table 1:** Baseline characteristics of the subjects

Variable	Nagoya *n* = 48	Maebashi *n* = 58	Total *n* = 106
Demographics			
Age, years	64.0 (52.0–70.8)	64.0 (52.0–71.0)	64.0 (52.0–71.0)
Sex, male, *n* (%)	41 (85.4)	46 (79.3)	87 (82.1)
Length of stay in hospital, days	28.0 (19.0–51.0)	32.5 (19.3–57.8)	29.0 (19.0–54.0)
Condition of cardiac arrest			
Witness, *n* (%)	39 (81.3)	49 (84.5)	88 (83.0)
Bystander, *n* (%)	29 (60.4)	32 (55.2)	61 (57.5)
Initial rhythm, shockable, *n* (%)	27 (56.3)	39 (67.2)	66 (62.3)
Duration of resuscitation effort, min	18.0 (12.5–28.5)	18.0 (8.0–28.0)	18.0 (10.0–28.8)
Presumed cardiac etiology, *n* (%)	29 (60.4)	38 (65.5)	67 (63.2)
GCS, *M* ≥ 2, *n* (%)^a^	29 (61.7)	38 (66.7)	67 (64.4)
pH^b^	7.07 ± 0.03	7.14 ± 0.03	7.11 ± 0.02
Time to initiation of targeted temperature management, hours	2.5 (1.5–3.0)	2.5 (2.0–3.0)	2.5 (1.5–3.0)
Time to targeted setting temperature, hours	4.5 (3.4–6.0)	5.0 (3.0–9.0)	5.0 (3.0–7.0)
Outcome			
Good (CPC ≤ 2), *n* (%)	21 (43.8)	24 (41.4)	45 (42.5)
Poor (CPC ≥ 3), *n* (%)	27 (56.2)	34 (58.6)	61 (57.5)

Data are presented as the median and interquartile ranges (25–75% percentile) or as absolute frequencies with percentages. Data are presented as mean ± standard error, as the median and interquartile ranges (25–75% percentile) or as absolute frequencies with percentages

*Nagoya* Nagoya University Hospital, *Maebashi* Japan Red Cross Maebashi Hospital, *GCS* Glasgow Coma Scale

^a^*n* = 2

^b^*n* = 2

The accuracies (sensitivity, specificity) of the emergency physicians’ and radiologists’ interpretation were (0.34, 1.00) and (0.41, 0.93), respectively. To evaluate these accuracies objectively, we calculated the GWRs and also examined the accuracy of the prediction using the GWR cutoff values of 1.16 and 1.13 (Table 2). The exact McNemar test showed no significant differences in either the sensitivity or the specificity between the two interpretations (sensitivity: *p* value = 0.34, specificity: *p* value = 0.25). Also, good conformity was confirmed between the two interpretations, with a calculated kappa statistic of 0.66 (95% CI 0.48–0.83, Table 3).

**Table 2:** Predictive accuracies of emergency physicians’ interpretation, radiologists’ interpretation, and the cutoff value of GWR < 1.16 and 1.13

	Interpreters		GWR < 1.16	GWR < 1.13
	By emergency physicians	By radiologists		
Sensitivity	0.34 (0.23–0.48)	0.41 (0.29–0.54)	0.54 (0.41–0.67)	0.28 (0.17–0.41)
Specificity	1.00 (0.92–1.00)	0.93 (0.82–0.99)	0.64 (0.49–0.78)	0.98 (0.88–1.00)

Data are presented as mean and 95% confidence interval

*GWR* gray matter attenuation to white matter attenuation ratio

**Table 3:** The conformity between these two interpretations

		Radiologists		
		Poor	Good	Total
Emergency physicians	Poor	17.0% (18/106)	2.8% (3/106)	21
	Good	9.4% (10/106)	70.8% (75/106)	85
	Total	28	78	106

Kappa statistics: 0.66 (95% CI 0.48–0.83). Data are presented as absolute frequencies with percentages

*95% CI* 95% confidence interval

## Discussion

Because of the shortage of radiologists in many countries, it is often not possible for radiologists to evaluate CT scans in real time at the ED [12, 13]. In such cases, emergency physicians have to interpret the images and manage the patients accordingly before the radiologists’ report becomes available. While several studies have compared the predictive accuracy of the interpretation by emergency physicians with that by the radiologists [17, 18], there was no study about the predictive accuracy of the interpretation of early brain CT by emergency physicians in PCAS patients who underwent TTM. Our study is the first study to examine the predictive accuracy of the interpretation by emergency physicians.

We investigated the predictive ability of the GWR using two cutoff values, as well as the predictive accuracies of the interpretations by emergency physicians and radiologists. GWR is one of the most reliable objective indices of hypoxic encephalopathy, and several studies (with small sample sizes) have reported the usefulness of calculation of the GWR for predicting the prognosis in PCAS patients using different cutoff points (1.10 to 1.20) [19–22]. In our study, we confirmed that the accuracy of poor prognosis prediction based on a GWR of < 1.13 was good, consistent with previous reports, while that based on a GWR of < 1.16 was inadequate. A study with a large sample size would be needed for detecting the best cutoff points.

In this study, the specificity of the emergency physicians’ interpretation for a poor prognosis sign was 1.00, which means that the likelihood of good recovery of the PCAS patients was extremely low if the emergency physicians interpreted the findings on early brain CT as being predictive of a poor prognosis. Previous study showed that a hypoxic encephalopathy sign on their brain CT was a reliable sign for a poor prognosis [9, 10], but few studies took into account whether the patients included in the study had undergone/not undergone TTM. Our study showed that, even if the PCAS patients underwent TTM, the predictive accuracy of the interpretation for poor prognosis was still high. From the viewpoint of cost-effectiveness, in patients in whom the emergency room physicians interpret the early brain CT findings in PCAS patients as being predictive of a poor prognosis, TTM may fail to be of benefit, although further studies are needed.

There were some limitations of this study. First, it was a retrospective study that involved a review of the electronic charts of the patients. The interpretations from their CT could have been biased by other information that could have influenced the prognosis, such as the clinical histories and physical examination findings of the patients. Second, the study was performed at only two participant hospitals, and further multicenter studies would be needed. It would be a great interest to conduct a prospective multicenter study to evaluate the accuracy of interpretation of early brain CT, obtained before the initiation of TTM, so as to optimize the management in patients with PCAS.

## Conclusions

The emergency physicians’ interpretation of the early brain CT findings in PCAS patients treated by TTM was as reliable as that of radiologists, in terms of prediction of the prognosis.

## Abbreviations

95% CI: 95% confidence interval
CPC: Cerebral Performance Categories
ED: Emergency department
GWR: Gray matter attenuation to white matter attenuation ratio
ICU: Intensive care unit
PCAS: Post-cardiac arrest syndrome
ROSC: Return of spontaneous circulation
TTM: Targeted temperature management
